# Comparing the effectiveness of different strains of *Wolbachia* for controlling chikungunya, dengue fever, and zika

**DOI:** 10.1371/journal.pntd.0006666

**Published:** 2018-07-30

**Authors:** Ling Xue, Xin Fang, James M. Hyman

**Affiliations:** 1 Department of Mathematics, Harbin Engineering University, Harbin, China; 2 Department of Mathematics, University of Manitoba, Canada; 3 Department of Mathematics, Tulane University, New Orleans, United States of America; Institute for Disease Modeling, UNITED STATES

## Abstract

Once *Aedes aegypti* and *Aedes albopictus* mosquitoes that spread Chikungunya virus, dengue virus, and Zika virus are infected with *Wolbachia*, they have reduced egg laying rates, reduced transmission abilities, and shorter lifespans. Since most infected mosquitoes are only infectious in the last few days of their lives, shortening a mosquito’s lifespan by a day or two can greatly reduce their abilities to spread mosquito-borne viral diseases, such as Chikungunya, dengue fever, and Zika. We developed a mathematical model to compare the effectiveness of the wMel and wAlbB strains of *Wolbachia* for controlling the spread of these viruses. The differences among the diseases, mosquitoes, and *Wolbachia* strains are captured by the model parameters for the mosquito-human transmission cycle. Moreover, the model accounts for the behavior changes of infectious population created by differences in the malaise caused by these viruses. We derived the effective and basic reproduction numbers for the model that are used to estimate the number of secondary infections from the infectious populations. In the same density of *Wolbachia*-free *Aedes aegypti* or *Aedes albopictus* mosquitoes, we observed that wMel and wAlbB strains of *Wolbachia* can reduce the transmission rates of these diseases effectively.

## Author summary

Mosquitoes infected with *Wolbachia* bacteria are less capable of transmitting dengue virus, Chikungunya virus, and Zika virus. We use a mathematical model to quantify the impact of infecting wild *Aedes aegypti* and *Aedes albopictus* mosquitoes with wAlbB and wMel strains of *Wolbachia* in reducing the transmission of these viruses. The model is a system of ordinary differential equations that accounts for reduced fitness of the *Wolbachia*-infected mosquitoes, reduced transmissibility of an infection, and the behavior changes of infected individuals caused by the infection. We derived an explicit formula for the effective reproduction number for when the host population are partially immune to new infections, as occurs in seasonal outbreaks. We compared the effectiveness of different species of mosquitoes, different strains of *Wolbachia*, and different diseases. Our model is a general model that can produce outputs for a specific location, once the data for the location are available to parameterize the model. The spatial heterogeneity of the populations must be considered before using this model to help guide policy decisions.

## Introduction

The current pandemics of Chikungunya caused by Chikungunya virus (CHIKV), dengue fever caused by dengue virus (DENV), and Zika resulted from Zika virus (ZIKV) infect over one hundred million people each year [1]. In the past decade, CHIKV has spread around the world [2, 3] and recently over a million cases occurred in the Caribbean and Latin America. Symptoms of infection with CHIKV include high fever and headache, with arthritis affecting joints, and may sustain for weeks or months [4]. Dengue fever has spread around the world and is endemic in South America and Asia [1]. People infected with DENV have symptoms ranging from mild headaches, severe headaches and joint pains to hemorrhagic or shock syndrome fever. Recently, ZIKV has spread through the Americas, starting with a 2015 explosive outbreak in Brazil. Although most people infected with ZIKV have mild symptoms, there is a correlation between infections in pregnant women and their children born with microcephaly (an abnormally small brain). Currently no vaccines are commercially available for Zika and Chikungunya. The first dengue vaccine, Dengvaxia (CYD-TDV), registered in Mexico in December, 2015 is not effective for the younger ones and for the seronegative population due to ethical concern of non-maleficence [5].

Although both *Aedes aegypti* (*Ae*. *aegypti*) and *Aedes albopictus* (*Ae*. *albopictus*) mosquitoes can transmit these viruses, *Ae*. *aegypti* mosquitoes are more abundant in urban areas and are the primary vectors. Current prophylactic measures include individual protection from mosquito bites, such as applying mosquito repellent and avoiding exposure to mosquitoes. Limited control options focus on reducing the mosquito populations, including spraying insecticides, treatments, and removal of mosquito breeding sites. These control strategies are hard to sustain because of the vigilance needed to eradicate the breeding sites, the expense of repeated spraying, and the mosquitoes developing resistance to the insecticides.

The cost and difficulty of eliminating the mosquitoes motivate the need of developing more efficient strategies to mitigate and control the transmission of these viruses. *Wolbachia* is a genus of bacteria that can infect 25-75% of all insects [6] and recent studies have shown that some strains of *Wolbachia* can increase the resistance of mosquitoes being infected with these viruses [7]. Recent experiments have shown that wMel strain of *Wolbachia* infection in *Ae*. *albopictus* inhibits the growth of CHIKV [8]. Moreover, *Wolbachia* infection in mosquitoes reduces egg laying rates, reduces their ability of transmitting viral infections, and shortens their lifespans by a few days. Since many mosquitoes infected with DENV, ZIKV, or CHIKV are infectious for only a few days at the end of their lifespans, the shortened lifespans of *Wolbachia*-infected mosquitoes result in more mosquitoes dying before they can transmit the infection. This implies that *Wolbachia*-infected mosquitoes sustaining in a wild mosquito population will be less likely to transmit these viral diseases.

Infected females can pass the bacteria to their offsprings and spread *Wolbachia* vertically from one generation to the next. *Wolbachia* disrupts the reproductive cycle of hosts through a cytoplasmic incompatibility between the sperms and eggs. Cytoplasmic incompatibility occurs when *Wolbachia*-infected male mosquitoes mate with *Wolbachia*-free female mosquitoes, and causes the *Wolbachia*-free females to produce fewer progeny [9, 10]. These effects provide the *Wolbachia* a vertical transmission advantage. However, this is offset by a reduced lifespan and reduced number of eggs hatched by a *Wolbachia*-infected mosquito.

When *Wolbachia*-infected mosquitoes are introduced in a wild population of uninfected mosquitoes, the infection is quickly wiped out unless the fraction of infected mosquitoes exceeds a threshold *θ* of the total population. Recent mathematical models have established these threshold conditions as *θ* = 0.15, 0.24, and 0.6 for wAlbB-, wMel-, and wMelPop-infected mosquitoes to establish a stable population, respectively [11]. Note that these threshold estimates are for an ideally controlled situation where mosquitoes do not mix with surrounding uninfected mosquitoes. The thresholds could be much higher for a release in the wild. Recent studies have found that maintaining a sustained wMelPop-infection requires continually introducing new wMelPop-infected mosquitoes into the wild population [12]. A recent research has reported that large-scale releases of *Wolbachia*-infected *Ae*. *aegypti* in the city of Cairns, Australia, invaded and spread through the populations, while *Wolbachia* infection at a smaller release site collapsed due to the immigration of *Wolbachia*-free mosquitoes from surrounding areas [13]. Population cage experiments indicated that the wAlbB strain can be successfully introduced into populations, and subsequently persist and spread [14]. Ndii et al. analyzed a first-order differential equation and found that a significant reduction in human dengue cases can be obtained by releasing wMel-infected mosquitoes, instead of wMelPop-infected mosquitoes due to the greatly reduced lifespans [15]. Ferguson et al. developed a mathematical model of DENV transmission incorporating the dynamics of viral infection in humans and mosquitoes, and predicted that wMel-infected *Ae*. *aegypti* mosquitoes have a substantial effect on transmission [16]. Ross et al. found that, in most situations, it was easier to establish wMel than wAlbB in mosquito populations, except when the conditions were particularly hot [17]. They also observed that the wMel infected larvae survived better than wAlbB infected larvae under starvation conditions [18].

Many mathematical models have been developed to explore conditions under which *Wolbachia* can be used to fight against the spread of viruses effectively. The analysis of a compartmental mathematical model showed that a significant reduction in human dengue cases can be obtained provided that *Wolbachia*-infected mosquitoes persist when competing with *Wolbachia*-free mosquitoes [15]. Zhang et al. developed an ordinary differential equation (ODE) model to assess how best to replace DENV vectors with *Wolbachia*-infected mosquito populations and the results showed that successful population replacement will rely on the selection of suitable strains of *Wolbachia* and careful design of augmentation methods [19]. The analysis for an impulsive model for *Wolbachia* infection control of mosquito-borne diseases with general birth and death rates showed that strategies may be different due to different birth and death rate functions, the type of *Wolbachia* strains, and the initial number of *Wolbachia*-infected mosquitoes [20]. Xue et al. [21] created a two-sex model that included an egg/aquatic stage for the mosquito lifecycle and observed that an endemic *Wolbachia* infection can be established only if a sufficient number of infected mosquitoes are released. Recently, this model was extended by Qu et al. [22] to better account for the cytoplasmic incompatibility by considering the fact that most female mosquitoes only mate once. They used the model to investigate the effectiveness of multiple releases of infected mosquitoes in sustaining an endemic *Wolbachia* infection.

Manore et al. [23] used a mathematical model to compare the spread of DENV and CHIKV in *Ae*. *aegypti* and *Ae*. *albopictus* mosquitoes that are not infected with *Wolbachia*. Our study is based on extending these results to evaluate the effectiveness of infecting these mosquitoes with different strains of *Wolbachia* to show their different roles in controlling different vector-borne diseases. In our model, we assume that lifespans of the infected adult mosquitoes are slightly shorter than those of uninfected mosquitoes (reducing transmission), and the larval survival rates of wAlbB-infected mosquitoes are less than those of wMel-infected mosquitoes (making invasion somewhat potentially harder for wAlbB). We evaluated the effectiveness of infecting these mosquitoes with wMel and wAlbB strains of *Wolbachia* to show their different roles in controlling the transmission of DENV, ZIKV, and CHIKV. Since transmission of ZIKV is estimated to be similar to transmission of DENV but exact values of parameters are not available [24], we assume that the parameter values for ZIKV are the same as those for DENV except the fraction of infectious humans exposed to mosquito bites. Our simulation results show that the differences between the spread of DENV and ZIKV lie in different behaviors of infectious humans, and wMel is more effective than wAlbB strain of *Wolbachia* in simulations with the available baseline parameters.

## Methods

**Fig 1:**
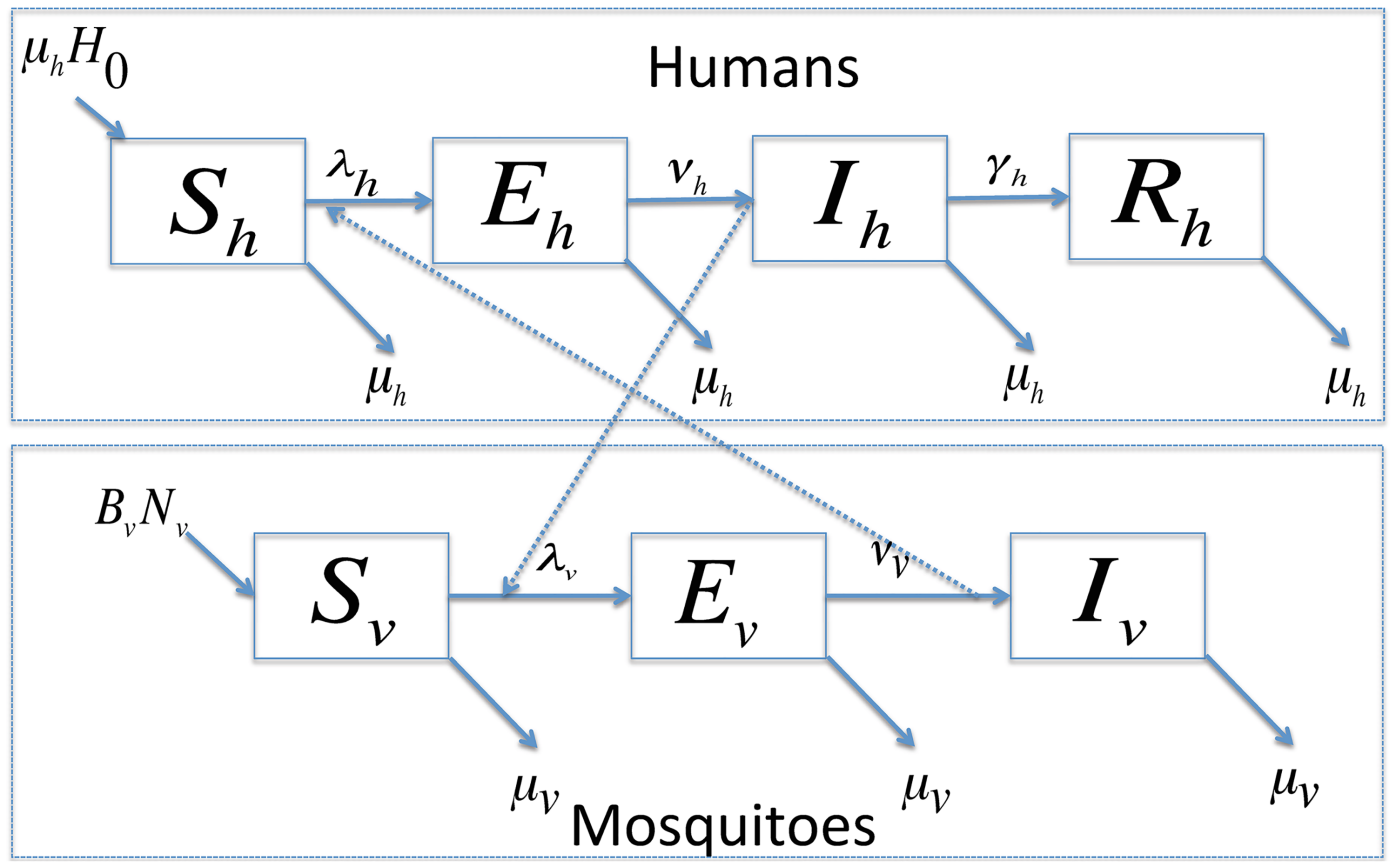
The schematic diagram for Model (1). Susceptible humans can be infected from bites of an infectious mosquito, and susceptible mosquitoes can be infected from blood meals on an infectious person. The forces of infection, λ_*_, are defined in Eq (4). After being infected, the susceptible individuals progress through the infectious states. Humans advance from the infectious state to the recovered state, while we assume that mosquitoes do not recover from an infection before dying.

Our compartmental model (Fig 1) divides the human population into four classes: susceptible, *S*_*h*_, exposed (infected but not infectious), *E*_*h*_, infectious, *I*_*h*_, and recovered (immune), *R*_*h*_, and splits the mosquitoes into three classes: susceptible, *S*_*v*_, exposed, *E*_*v*_, and infectious, *I*_*v*_. We assume that humans advance from the infectious state to the recovered state, while mosquitoes do not. Model parameters are defined in Table 1, and the ODEs are:

$$\begin{array}{c} \begin{array}{cc} \frac{dS_{h}}{dt} & =\mu_{h}H_{0}-\alpha_{v}\left(t\right)I_{v}-\mu_{h}S_{h},\\ \frac{dE_{h}}{dt} & =\alpha_{v}\left(t\right)I_{v}-\nu_{h}E_{h}-\mu_{h}E_{h},\\ \frac{dI_{h}}{dt} & =\nu_{h}E_{h}-\gamma_{h}I_{h}-\mu_{h}I_{h},\\ \frac{dR_{h}}{dt} & =\gamma_{h}I_{h}-\mu_{h}R_{h},\\ \frac{dS_{v}}{dt} & =B_{v}\left(N_{v}\right)N_{v}-\alpha_{h}\left(t\right)I_{h}-\mu_{v}S_{v},\\ \frac{dE_{v}}{dt} & =\alpha_{h}\left(t\right)I_{h}-\nu_{v}E_{v}-\mu_{v}E_{v},\\ \frac{dI_{v}}{dt} & =\nu_{v}E_{v}-\mu_{v}I_{v}. \end{array} \end{array}$$ (1)

The equations are homogenous with respect to the populations of humans and mosquitoes. The solution is invariant as long as both populations are scaled by the same factors and the ratio *ρ*_*vh*_ = *V*_0_/*H*_0_ is kept fixed. That is, all the results in this study hold for other populations with the same vector-to-human ratio, *ρ*_*vh*_.

**Table 1:** Parameters for the Model (1).

Parameter	Description	Unit
*S*_*h*_(*t*)	The number of susceptible humans at time *t*	Number
*E*_*h*_(*t*)	The number of exposed humans at time *t*	Number
*I*_*h*_(*t*)	The number of infectious humans at time *t*	Number
*R*_*h*_(*t*)	The number of recovered humans at time *t*	Number
*S*_*v*_(*t*)	The number of susceptible mosquitoes at time *t*	Number
*E*_*v*_(*t*)	The number of exposed mosquitoes at time *t*	Number
*I*_*v*_(*t*)	The number of infectious mosquitoes at time *t*	Number
*H*_0_	The steady-state human population	Number
*b*(*t*)	The total number of bites per day	Bites Day^−1^
*b*_*iv*_(*t*)	The average number of bites per day for an infectious mosquito	Bites Day^−1^
*b*_*ih*_(*t*)	The total number of bites per day for an infected human	Bites Day^−1^
*R*_*hv*_(*t*)	The effective reproduction number for transmission from mosquitoes to humans	Dimensionless
*R*_*vh*_(*t*)	The effective reproduction number for transmission from humans to mosquitoes	Dimensionless
*ψ*_*v*_	Per capita birth rate of mosquitoes	Number Day^−1^
*σ*_*v*_	Average number of times one mosquito bites a human per day	Bites Day^−1^
*σ*_*h*_	The maximum number of mosquito bites a human tolerates per unit time	Bites Day^−1^
*β*_*hv*_	Probability of pathogen transmission from an infectious mosquito to a susceptible human per bite	Dimensionless
*β*_*vh*_	Probability of pathogen transmission from an infectious human to a susceptible mosquito per bite	Dimensionless
*ν*_*h*_	Average incubation rate for humans	Day^−1^
*ν*_*v*_	Average incubation rate for mosquitoes	Day^−1^
*γ*_*h*_	Per capita recovery rate for humans from the infectious state to the recovered state	Day^−1^
*μ*_*h*_	Per capita recruitment / death / emigration rate for humans	Day^−1^
*μ*_*v*_	Death rate for mosquitoes	Day^−1^
*K*_*v*_	Mosquito carrying capacity and steady-state	Number
*ϕ*_*ψ*_	Factor for decreased birth rate: $\psi_{v}=\phi_{\psi }{\tilde{\psi }}_{v}$	Dimensionless
*ϕ*_*μ*_	Factor for increased death rate: $\mu_{v}=\phi_{\mu }{\tilde{\mu }}_{v}$	Dimensionless
*ϕ*_*b*_	Factor for decreased biting rate: $\sigma_{v}=\phi_{b}{\tilde{\sigma }}_{v}$	Dimensionless
*ϕ*_*β*_	Factor for decreased transmissibility: $\beta_{hv}=\phi_{\beta }{\tilde{\beta }}_{hv}$	Dimensionless

Humans and mosquitoes leave the population through combined per capita recruitment / death / emigration rates, *μ*_*h*_ and *μ*_*v*_, respectively. Although the model equations can accommodate variations in the human and mosquito populations, we assume a constant size of human population in simulations. Susceptible humans enter the population at a fixed rate of *μ*_*h*_*H*_0_ per day to maintain a steady at-risk human population of *H*_0_. In our simulations, we assume that 1/*μ*_*h*_ = 20 years, where about 5% of the population turns over each year to account for individuals moving in and out of the population. Note that *μ*_*h*_ depends on the particular region being modeled and will be larger in regions where there are migrant workers, or smaller in isolated villages.

Mosquitoes are born into the population at the rate of *B*_*v*_(*N*_*v*_)*N*_*v*_ per day, where the mosquito birth and population saturation function *B*_*v*_ is given by [23]

$$B_{v}\left(N_{v}\right)=\psi_{v}-\left(\psi_{v}-\mu_{v}\right)\frac{N_{v}}{V_{0}}.$$

Here *ψ*_*v*_ is the mosquito birth rate when there are abundant resources for the eggs and larvae, and *V*_0_ is the carrying capacity and steady state for the mosquitoes.

The viruses can be transmitted from infectious mosquitoes to susceptible humans, and from infectious humans to susceptible mosquitoes through blood meals. We formulate the viral transmission in terms of the rate at which infectious mosquitoes or infectious humans infect others, rather than the traditional formulation in terms of the rate at which the susceptible individuals are being infected as in [23]. The notation in this infectious viewpoint emphasizes that the infectious population drive the epidemic and clarifies the derivation for effective reproduction number.

The force from infection for humans, *α*_*v*_(*t*), and force from infection for mosquitoes, *α*_*h*_(*t*), are the rates at which infectious individuals infect others and are equal to the product

$$\begin{array}{cc} \alpha_{*}\left(t\right)= & \;\;\;\;(\text{probability}\;\text{of}\;\text{transmission}\;\text{per}\;\text{bite,}\;\beta_{*})\\ & \;\times (\text{number}\;\text{of}\;\text{bites}\;\text{per}\;\text{infectious}\;\text{individual}\;\text{per}\;\text{day},\;b_{i*})\\ & \;\times (\text{probability}\;\text{that}\;\text{an}\;\text{infectious}\;\text{individual}\;\text{bites}\;\text{a}\;\text{susceptible}\;\text{individual,}\;P_{s*}), \end{array}$$

where * = *v* or *h*. That is,

$$\begin{array}{c} \begin{array}{cc} \alpha_{v}\left(t\right) & =\beta_{hv}b_{iv}\left(t\right)P_{sh}\left(t\right),\\ \alpha_{h}\left(t\right) & =\beta_{vh}b_{ih}\left(t\right)P_{sv}\left(t\right). \end{array} \end{array}$$ (2)

Here *β*_*hv*_ is the probability that an infectious mosquito will infect a susceptible human in one bite. Similarly, *β*_*vh*_ is the probability that an infectious human will infect a susceptible mosquito in one bite.

The parameter *b*_*iv*_ is the average number of times an infectious mosquito bites a susceptible human per day. Similarly, *b*_*ih*_ is the average number of times that an infectious human is bitten by a susceptible mosquito per day. We assume that the biting rates for the mosquitoes do not change after they become infected.

We assume that infectious people, especially those with symptoms may avoid exposure to mosquito bites. Around 20–93% individuals infected with DENV are asymptomatic [25, 26], and asymptomatic prevalence of Chikungunya was estimated in the range 16.7–27.7% during some recorded outbreaks [27, 28], while only around 20% of people infectious with ZIKV have significant symptoms [29–31]. The biting rate accounts for a fraction, *π*, of infectious humans do not change their behaviors due to the illnesses and continue being bitten by mosquitoes at the same rate as the susceptible population. In our model, we assume *π* = 0.75 for DENV, *π* = 0.3 for CHIKV, and *π* = 0.8 for ZIKV. The remaining fraction (1 − *π*) avoid being bitten by mosquitoes. This behavior change has a significant impact on the force of infection from humans to mosquitoes and is an important aspect of any vector-borne transmission model.

We define *σ*_*v*_ as the maximum rate at which a typical mosquito will bite humans per day, and *σ*_*h*_ is the maximum number of bites that a susceptible human will tolerate being bitten per day. We define *N*_*v*_ = *S*_*v*_ + *E*_*v*_ + *I*_*v*_ as the number of mosquitoes that bite humans. Similarly, we define *N*_*h*_ = *S*_*h*_ + *E*_*h*_ + *πI*_*h*_ + *R*_*h*_ as the number of humans being bitten by mosquitoes. Recall that the at-risk population, *N*_*h*_, is only a fraction of the total human population since some infectious people are not being bitten by mosquitoes. One of the most difficult parameters to estimate when applying any vector-borne epidemic models to a particular situation is the fraction of the population at risk of an infection. Using these definitions, *σ*_*v*_*N*_*v*_ is the maximum number of bites a mosquito seeks per day, while the maximum number of available human bites per day is *σ*_*h*_*N*_*h*_.

The total number of times that all the mosquitoes bite humans must equal to the total number of times that humans are bitten by mosquitoes. To enforce this balance condition, we extend the harmonic average described in [23, 32] and define the total number of bites per day (total biting rate) as

$$b\left(t\right)=\frac{\sigma_{v}N_{v}\sigma_{h}N_{h}}{\sigma_{v}N_{v}+\sigma_{h}N_{h}}.$$ (3)

This biting rate allows a wide range of vector-to-host ratios, as opposed to the more standard frequency-dependent contact rates that are applicable only over a limited range of vector-to-host ratios [33].

The total number of bites from mosquitoes is *b*(*t*) = *b*_*v*_*N*_*v*_, where *b*_*v*_ is the average number of bites per mosquito per day (the biting rate). Because we assume that the infection does not affect the biting rate, the average number of bites per day for an infectious mosquito is also *b*_*iv*_ = *b*_*v*_ = *b*(*t*)/*N*_*v*_. To satisfy the balance condition, the total number of bites on humans is also *b*(*t*) = *b*_*h*_ *N*_*h*_, where *b*_*h*_ is the average number of times an infectious human being bitten per day. Because (1 − *π*) of infectious humans have changed their behaviors and are not being bitten, the average number of times an infectious human being bitten per day is *b*_*ih*_ = *πb*_*h*_ = *πb*(*t*)/*N*_*h*_.

We define *P*_*sh*_(*t*) as the probability that an infectious mosquito bites a susceptible human. If we assume that the bites on humans are randomly distributed, then *P*_*sh*_(*t*) = *S*_*h*_(*t*)/*N*_*h*_(*t*). Similarly, *P*_*sv*_(*t*) is the probability that when an infectious human is bitten, the bite is from a susceptible mosquito. Hence, *P*_*sv*_(*t*) = *S*_*v*_(*t*)/*N*_*v*_(*t*).

The ODES in Model (1) are formulated from the viewpoint of infectious population where the transmission parameter, *α*, is the force *from* infection. This is equivalent to the usual way of formulating the equations for force *of* infection from the susceptible viewpoint [23]. From the susceptible viewpoint, the ODEs for the susceptible humans and vectors have the form:

$$\begin{array}{c} \begin{array}{cc} \frac{dS_{h}}{dt} & =\mu_{h}H_{0}-\lambda_{h}\left(t\right)S_{h}-\mu_{h}S_{h},\\ \frac{dS_{v}}{dt} & =B_{v}\left(N_{v}\right)N_{v}-\lambda_{v}\left(t\right)S_{v}-\mu_{v}S_{v}. \end{array} \end{array}$$

Here λ is force of infection and is related to *α* by

$$\begin{array}{ll} \lambda_{h}\left(t\right)S_{h}\left(t\right)=\alpha_{v}\left(t\right)I_{v}\left(t\right), & \lambda_{h}\left(t\right)=\beta_{hv}b_{sh}\left(t\right)P_{iv}\left(t\right),\\ \lambda_{v}\left(t\right)S_{v}\left(t\right)=\alpha_{h}\left(t\right)I_{h}\left(t\right), & \lambda_{v}\left(t\right)=\beta_{vh}b_{sv}\left(t\right)P_{ih}\left(t\right), \end{array}$$ (4)

where the factors for λ_*_ are all from the viewpoint of the susceptible population, instead of the viewpoint of the infectious population in Eq (2). *P*_*ih*_(*t*) = *πI*_*h*_(*t*)/*N*_*h*_(*t*) is the probability that when a susceptible mosquito bites a human, the human is infectious; *b*_*sh*_ = *b*(*t*)/*N*_*h*_ is the rate at which susceptible humans are bitten; *P*_*iv*_(*t*) = *I*_*v*_(*t*)/*N*_*v*_(*t*) is the probability that when a susceptible human is bitten, the mosquito is infectious; and *b*_*sv*_ = *b*(*t*)/*N*_*v*_ is the rate at which susceptible mosquitoes bite humans.

### The effective reproduction number

The basic reproduction number, $R_{0}$, is defined as the number of new infections produced by one infected individual in a completely susceptible population. When the population is not fully susceptible, or more than one person is infected, then the effective reproduction number, $R_{eff}\left(t\right)$, estimates the number of secondary cases produced by a typical infected individual at any time during the epidemic. We derived the effective reproduction number from infectious point of view for DENV, ZIKV, and CHIKV to estimate the reproduction rate of an epidemic at any stage.

Because this is a bipartite transmission cycle, mosquitoes only infect humans and humans only infect mosquitoes, we have different effective reproduction numbers for each part of the cycle. We define $R_{hv}\left(t\right)$ as the effective reproduction number for transmission from mosquitoes to humans, and is the average number of humans infected by one infectious mosquito. Similarly, $R_{vh}\left(t\right)$ defined as the effective reproduction number for transmission from humans to mosquitoes, is the average number of mosquitoes infected by one infectious human. These dimensionless numbers are defined by

$$R_{hv}\left(t\right)=P_{v}\alpha_{v}\left(t\right)\tau_{iv},$$ (5)

$$R_{vh}\left(t\right)=P_{h}\alpha_{h}\left(t\right)\tau_{ih}.$$ (6)

Here, in Eq (5), *P*_*v*_ = *ν*_*v*_/(*ν*_*v*_ + *μ*_*v*_) is the probability that an infected mosquito survives through the incubation period and becomes infectious, *α*_*v*_(*t*) is the average number of susceptible people infected by an infectious mosquito per day, and *τ*_*iv*_ = 1/*μ*_*v*_ is the average life span of a mosquito. Similarly for Eq (6), *P*_*h*_ = *ν*_*h*_/(*ν*_*h*_ + *μ*_*h*_) is the probability that an infected human becomes infectious, *α*_*h*_(*t*) is the average number of susceptible mosquitoes infected by an infectious person per day, and *τ*_*ih*_ = 1/(*γ*_*h*_ + *μ*_*h*_) is the average time that a human remains infectious.

The explicit expressions of $R_{hv}\left(t\right)$ and $R_{vh}\left(t\right)$ are:

$$\begin{array}{cc} R_{hv}\left(t\right) & =\frac{\nu_{v}}{\left(\nu_{v}+\mu_{v}\right)}\beta_{hv}b_{iv}\frac{S_{h}\left(t\right)}{N_{v}\left(t\right)}\frac{1}{\mu_{v}},\\ R_{vh}\left(t\right) & =\frac{\nu_{h}}{\left(\nu_{h}+\mu_{h}\right)}\beta_{vh}b_{ih}\frac{S_{v}\left(t\right)}{N_{h}\left(t\right)}\frac{1}{\gamma_{h}+\mu_{h}}. \end{array}$$

Because a full transmission cycle is consisted of two stages, and $R_{eff}\left(t\right)$ measures the average effective reproduction number over one cycle. We take the geometric average of these two reproductive numbers to define:

$$R_{eff}\left(t\right)=\sqrt{R_{hv}\left(t\right)R_{vh}\left(t\right)}.$$

We denote $S_{h}^{*}$ as the population of susceptible people at the endemic equilibrium (EE) for Model (1). We define the fraction of humans susceptible at the EE, $S_{h}^{*}/H_{0}$ of the population has never been infected, as susceptibility of humans at EE,

$$\frac{S_{h}^{*}}{H_{0}}=1-\left(1-\frac{\pi }{R_{0}^{2}}\right)\frac{\nu_{v}\sigma_{v}\sigma_{h}\beta_{hv}V_{0}}{\nu_{v}\sigma_{v}\sigma_{h}\beta_{hv}V_{0}+\mu_{h}\left(\mu_{v}+\nu_{v}\right)\left(\sigma_{h}H_{0}+\sigma_{v}V_{0}\right)}.$$

To quantify the differences in impact of different strains of *Wolbachia* on an epidemic, we define the coefficient for effectiveness [15] as the relative decrease in the number of people predicted to be infected if the mosquitoes are infected with *Wolbachia*, *H*_*W*_, compared with the predicted number of people who will be infected if the mosquitoes are *Wolbachia*-free, *H*_*F*_;

$$\kappa =\frac{H_{F}-H_{W}}{H_{F}}=1-\frac{H_{W}}{H_{F}}.$$ (7)

If *κ* = 1, then *Wolbachia* is predicted to be effective in stopping all the infections, while if *κ* = 0, then it is predicted to have no effects on the epidemic.

### The basic reproduction number

The basic reproduction number is the effective reproduction number at the disease free equilibrium where *S*_*v*_(0) = *V*_0_ and *S*_*h*_(0) = *H*_0_, and all other states are zero:

$$R_{0}=\sqrt{R_{hv}\left(0\right)R_{vh}\left(0\right)}.$$

$R_{hv}\left(0\right)$ and $R_{vh}\left(0\right)$ are the effective reproduction numbers for the vectors and humans at the disease free equilibrium:

$$\begin{array}{cc} R_{hv}\left(0\right) & =\frac{\nu_{v}}{\left(\nu_{v}+\mu_{v}\right)}\beta_{hv}b_{iv}\left(0\right)\frac{1}{\mu_{v}},\\ R_{vh}\left(0\right) & =\frac{\nu_{h}}{\left(\nu_{h}+\mu_{h}\right)}\beta_{vh}b_{ih}\left(0\right)\frac{1}{\gamma_{h}+\mu_{h}}. \end{array}$$

The biting rates at the disease free equilibrium are *b*_*iv*_(0) = *b*(0)/*V*_0_ and *b*_*ih*_(0) = *πb*(0)/*H*_0_, where

$$b(0)=\frac{\sigma_{v}V_{0}\sigma_{h}H_{0}}{\sigma_{v}V_{0}+\sigma_{h}H_{0}}.$$

The basic reproduction number derived in this way is consistent with the $R_{0}$ computed using the next generation matrix approach in [23].

After an epidemic has run its course and the infection has died out, then the previously infectious people are immune to new infections. $R_{eff}$ is the average number of new infectious individuals produced in one cycle when an infectious human or mosquito is introduced into the population where some of the population is immune to infection. We define *p*_*h*_ = *R*_*h*_(0)/*H*_0_ as the fraction of people who are immune to the infection, such as those who have already had the disease or have been immunized. If we reinitialize our model at *t* = 0 with an infection-free equilibrium, such as the beginning of a seasonal outbreak, where *p*_*h*_ of the humans are immune to the infection, *R*_*h*_(0) ≠ 0, and *S*_*h*_(0) + *R*_*h*_(0) = *H*_0_. For this case, the effective reproduction number for human-to-mosquito transmission, $R_{vh}{\left(0\right)}_{eff}=R_{vh}\left(0\right)$, is unchanged, while the effective reproduction number for mosquito-to-human transmission is reduced to $R_{hv}{\left(0\right)}_{eff}=R_{hv}\left(0\right)\left(1-p_{h}\right)$. Therefore, the effective reproduction number for Model (1) with *p*_*h*_ people immune to infection becomes

$$R_{eff}\left(0\right)=\sqrt{R_{hv}{\left(0\right)}_{eff}R_{vh}{\left(0\right)}_{eff}}=\sqrt{R_{hv}\left(0\right)R_{vh}\left(0\right)\left(1-p_{h}\right)}=R_{0}\;\sqrt{1-p_{h}}.$$

Note that, unlike human-to-human transmitted disease where the $R_{0}$ is reduced by (1 − *p*_*h*_) when *p*_*h*_ of the population is immune, $R_{0}$ is only reduced by $\sqrt{1-p_{h}}$ in this bipartite epidemic.

### Model parameters

In the simulations, the parameters are set to the baseline values in Table 2, unless specifically stated otherwise. Most of the parameter values used in this study were derived, or extensively referenced, by Manore et al. [23] for disease transmission in *Wolbachia*-free mosquitoes. Manore et al. provided a comprehensive sensitivity analysis on how the model predictions change with respect to variations in the key parameters [23]. Although these baseline values are our best estimates for the parameters, they are scalar estimates from a distribution of possible values. To help quantify the uncertainty in the parameters, we will investigate the behavior of the model over a wide range of feasible parameters. The model predictions for a specific value of the basic reproduction number or the fraction of the population infected at the endemic equilibrium depend on the specific values used in the simulations. Although these specific values for these predictions are sensitive to the parameter values, we find that the qualitative differences between different diseases and strains of *Wolbachia* are fairly insensitive over the feasible ranges of parameters.

**Table 2:** The parameters for Model (1) with baseline values, range, and references. The behavior change parameter *π* = 0.75 for DENV, *π* = 0.3 for CHIKV, and *π* = 0.8 for ZIKV [25–31]. Time is in days, and the default baseline populations are *H*_0_ = 10,000 and *V*_0_ = 200,000 with 20 mosquitoes per human, *ρ*_*vh*_ = *V*_0_/*H*_0_ = 20. The model predictions scale to other populations with the same *ρ*_*vh*_.

Par	Baseline	Range	Reference	Par	Baseline	Range	Reference
DENV and ZIKV				CHIKV			
*Human*				*Human*			
*σ*_*h*_	19	0.1 − 50	[38]	*σ*_*h*_	19	0.1 − 50	[38]
1/*ν*_*h*_	5	4 − 7	[39, 40]	1/*ν*_*h*_	3	2 − 4	[4, 41–43]
1/*γ*_*h*_	6	4 − 12	[44, 45]	1/*γ*_*h*_	6	3 − 7	[43, 46]
1/*μ*_*h*_	20 × 365	(15 − 25) × 365	Assume	1/*μ*_*h*_	20 × 365	(15 − 25) × 365	Assume
DENV and ZIKV (*Ae*. *aegypti*)				CHIKV (*Ae*. *aegypti*)			
*β*_*hv*_	0.33	0.10 − 0.75	[47, 48]	*β*_*hv*_	0.24	0.001 − 0.35	[4, 49, 50]
*β*_*vh*_	0.33*ϕ*_*β*_	(0.10 − 0.75)*ϕ*_*β*_	[47, 48]	*β*_*vh*_	0.24*ϕ*_*β*_	(0.005 − 0.35)*ϕ*_*β*_	[4, 49, 50]
*ψ*_*v*_	0.30*ϕ*_*ψ*_	(0.28 − 0.32)*ϕ*_*ψ*_	[38, 51, 52]	*ψ*_*v*_	0.30*ϕ*_*ψ*_	(0.28 − 0.32)*ϕ*_*ψ*_	[38, 51, 52]
*σ*_*v*_	0.5*ϕ*_*b*_	(0.33 − 1)*ϕ*_*b*_	[53, 54]	*σ*_*v*_	0.5*ϕ*_*b*_	(0.33 − 1)*ϕ*_*b*_	[53, 54]
1/*ν*_*v*_	10	7 − 14	[39, 55]	1/*ν*_*v*_	11	7 − 15	[49]
1/*μ*_*v*_	14/*ϕ*_*μ*_	(8 − 42)/*ϕ*_*μ*_	[53, 56, 57]	1/*μ*_*v*_	14/*ϕ*_*μ*_	(8 − 42)/*ϕ*_*μ*_	[53, 56, 57]
DENV and ZIKV (*Ae*. *albopictus*)				CHIKV (*Ae*. *albopictus*)			
*β*_*hv*_	0.31	0.1 − 0.5	[48, 58]	*β*_*hv*_	0.33	0.001 − 0.54	[41, 50, 59]
*β*_*vh*_	0.31*ϕ*_*β*_	(0.1 − 0.5)*ϕ*_*β*_	[48, 58]	*β*_*vh*_	0.33*ϕ*_*β*_	(0.3 − 0.9)*ϕ*_*β*_	[49, 50, 59, 60]
*ψ*_*v*_	0.24*ϕ*_*ψ*_	(0.22 − 0.26)*ϕ*_*ψ*_	[38, 51, 61]	*ψ*_*v*_	0.24*ϕ*_*ψ*_	(0.22 − 0.26)*ϕ*_*ψ*_	[38, 51, 61]
*σ*_*v*_	0.26*ϕ*_*b*_	(0.19 − 0.39)*ϕ*_*b*_	[62, 63]	*σ*_*v*_	0.26*ϕ*_*b*_	(0.19 − 0.39)*ϕ*_*b*_	[62, 63]
1/*ν*_*v*_	10	7 − 14	[51]	1/*ν*_*v*_	3.5	2 − 6	[41, 46, 64–66]
1/*μ*_*v*_	21/*ϕ*_*μ*_	(14 − 42)/*ϕ*_*μ*_	[41, 42, 51]	1/*μ*_*v*_	21/*ϕ*_*μ*_	(14 − 42)/*ϕ*_*μ*_	[41–43, 46]

We assume that the probability of transmission per bite from a mosquito to a human is related to the viral load in the mosquito. Recent experimental comparisons of the growth of DENV, ZIKV, and CHIKV in mosquitoes indicate that the viral loads and the extrinsic incubation period (EIP) for an infected mosquito to become infectious are comparable [34]. Because there are no experimental estimates for the infectivity of ZIKV-infected mosquitoes, we assume that the parameter values for infectivity of ZIKV are the same as those of DENV in our simulations. Note that, although we assume that the probability of transmission per mosquito bite is assumed to be the same for ZIKV- and DENV- infected mosquitoes. The behavior changes of humans infectious with ZIKV and DENV are different, leading to very different epidemics. That is, one must be very careful in extrapolating findings between ZIKV and DENV epidemics [24].

Duong et al. [26] showed that asymptomatic individuals infected with DENV may be infectious before the onset of symptoms and continue infecting mosquitoes as they visit multiple locations during the day. They also noted that sick people who are hospitalized or stay at home are only exposed to their residential mosquitoes. Grange et al. [25] summarized data from a large number of studies, showing that often 20–93% of DENV infected individuals are asymptomatic. In our simulations, we assume that 75% of DENV-infectious people continue exposing to mosquitoes (*π* = 0.75).

Bloch et al. [27] concluded that about 62.5% CHIKV infections are symptomatic through extensive statistical analysis. They observed that about one-third of CHIKV-infected participants are asymptomatic, which is consistent with estimates of 3–39% asymptomatic cases in past outbreaks. Robinson et al. [28] also noted that 16.7–27.7% of the infections in Chikungunya outbreaks are asymptomatic. In our simulations, we assume that 30% of infectious people with CHIKV continue exposing to mosquitoes (*π* = 0.30).

ZIKV infection is a self-limiting illness that is mostly asymptomatic. Lazear et al. [29] noted that approximately 20% of the individuals infected with ZIKV progress to a clinically apparent febrile illness, although rarely hospitalized. Rajah et al. [30] also observed that 20% of the people infected with ZIKV present mild symptoms. In our simulations, we assume that 80% of ZIKV-infectious people continue exposing to mosquitoes (*π* = 0.80).

The *Wolbachia* infection changes the mosquito’s birth, death, biting rates, and the transmissibility of an infection. We account for the change in these parameters by including a scaling factor, ϕ_*_. We identify the rates of *Wolbachia*-free mosquitoes by a tilde, $\tilde{\cdot }$, and define the factors for *Wolbachia*-infected mosquitoes as:

*ϕ*_*ψ*_—factor for decreased birth rate: $\psi_{v}=\phi_{\psi }{\tilde{\psi }}_{v},$
*ϕ*_*μ*_—factor for increased death rate: $\mu_{v}=\phi_{\mu }{\tilde{\mu }}_{v},$
*ϕ*_*β*_—factor for decreased transmissibility: $\beta_{hv}=\phi_{\beta }{\tilde{\beta }}_{hv},$
*ϕ*_*b*_—factor for decreased biting rate: $\sigma_{v}=\phi_{b}{\tilde{\sigma }}_{v}.$

The values for the factors ϕ_*_ in Table 3 are used in Table 2 for the baseline parameter values used for this study. These factors coincide with the factors applied by [35] for comparing the effects of different strains of *Wolbachia*. The ranges of the lifespans for wMel-infected, wAlbB-infected, and *Wolbachia*-free mosquitoes in Table 2 coincide with the plot for longevity of wAlbB- and wMel- infected mosquitoes plotted by Joubert et al. [36].

**Table 3:** The scaling factors for the *Wolbachia*-free model parameters to convert some parameters to their appropriate values for *Wolbachia*-infected mosquitoes. *ϕ*_*ψ*_—factor for decreased birth rate: $\psi_{v}=\phi_{\psi }{\tilde{\psi }}_{v}$, *ϕ*_*μ*_—factor for increased death rate: $\mu_{v}=\phi_{\mu }{\tilde{\mu }}_{v}$, *ϕ*_*β*_—factor for decreased transmissibility: $\beta_{hv}=\phi_{\beta }{\tilde{\beta }}_{hv}$, *ϕ*_*b*_—factor for decreased biting rate: $\sigma_{v}=\phi_{b}{\tilde{\sigma }}_{v}$.

*Wolbachia* strain	*ϕ*_*ψ*_	*ϕ*_*μ*_	*ϕ*_*β*_	*ϕ*_*b*_
wAlbB	0.85 [11, 35, 67]	1.00 [11, 35, 68]	0.63 [11, 68]	1.00
wMel	0.95 [11, 35, 69]	1.10 [11, 35, 69]	0.50 [69]	0.95
*Wolbachia*-free	1.00	1.00	1.00	1.00

## Results

We compare the differences in the spread of DENV, ZIKV, and CHIKV in wMel- and wAlbB-infected *Ae*. *aegypti* and *Ae*. *albopictus* mosquitoes with the spread in *Wolbachia*-free mosquitoes. In the simulations, the parameters are set to the baseline values in Table 2, unless specifically stated otherwise. The baseline values are the best estimates available for these parameter values. Since parameter uncertainty exists, it is important to investigate the behavior of the model over the wide range of feasible parameters. By investigating the model over the full range of parameters, we have focused on the qualitative differences between different infections and strains of *Wolbachia*.

### Cumulative number of infectious humans

**Fig 2:**
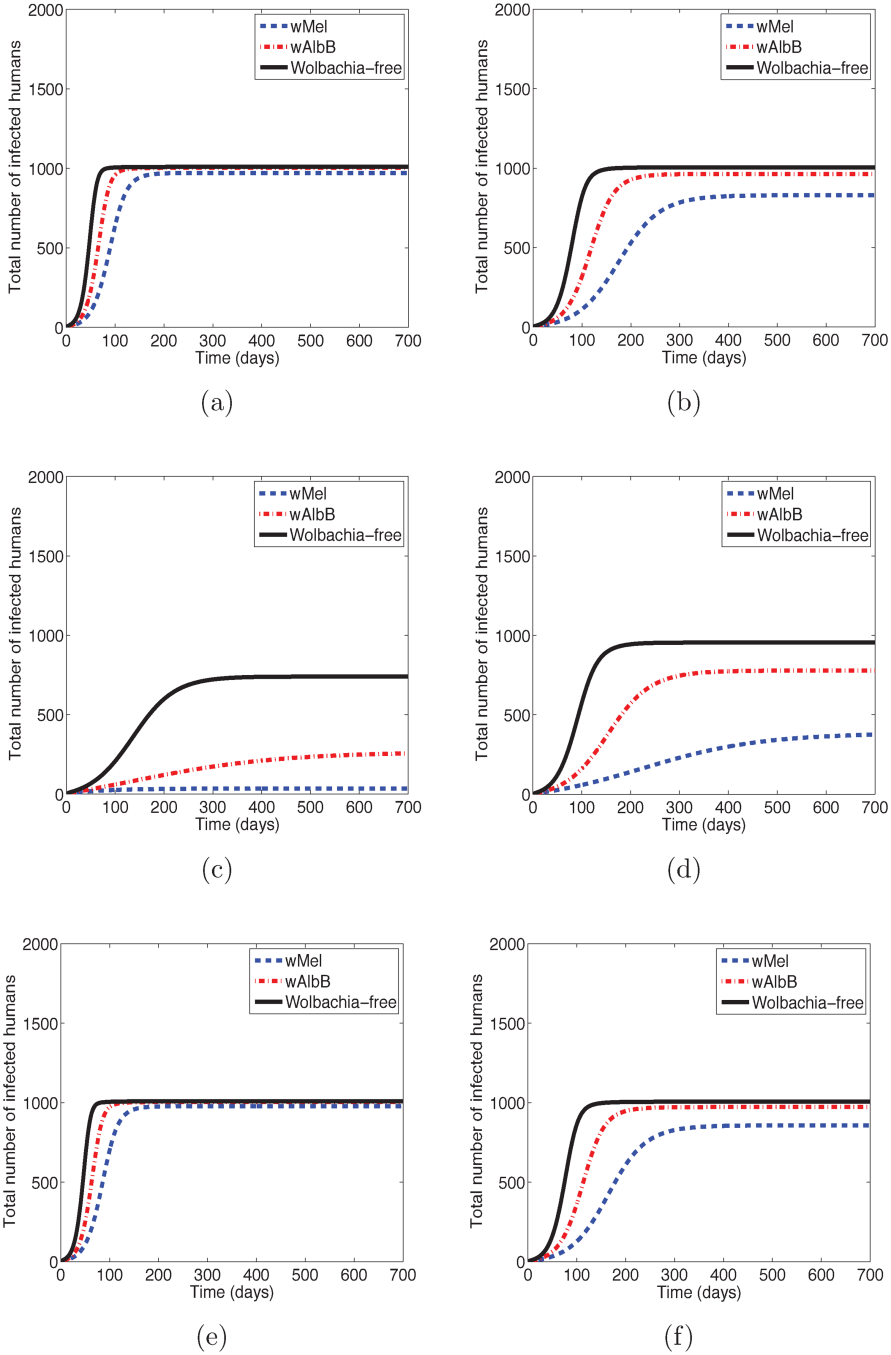
The total number of infectious humans produced by infectious *Ae*. *aegypti* and *Ae*. *albopictus* for the baseline parameters in Table 2. The model predicts that the wMel-infected mosquitoes are more effective than wAlbB-infected mosquitoes in reducing the number of people infectious with the diseases. We find that for the same mosquito densities, *Ae*. *aegypti* is more effective than *Ae*. *albopictus* in transmitting DENV and ZIKV, while the opposite is true for CHIKV. (a) DENV infection transmitted by *Ae*. *aegypti*. (b) DENV infection transmitted by *Ae*. *albopictus*. (c) CHIKV infection transmitted by *Ae*. *aegypti*. (d) CHIKV infection transmitted by *Ae*. *albopictus*. (e) ZIKV infection transmitted by *Ae*. *aegypti*. (f) ZIKV infection transmitted by *Ae*. *albopictus*.

Fig 2 shows the cumulative number of infectious humans up to time *t* = 700 when 0.1% of humans are infected at *t* = 0. The figure illustrates that the wMel-infected mosquitoes are more effective in slowing disease transmission than wAlbB-infected mosquitoes. For DENV and ZIKV, the number of people infected by *Ae*. *aegypti* mosquitoes is greater than the number of humans infected by *Ae*. *albopictus* mosquitoes carrying the same strain of *Wolbachia*. The opposite is true for CHIKV where the number of people infected by *Ae*. *albopictus* mosquitoes is greater than the number of people infected by *Ae*. *aegypti* mosquitoes carrying the same strain of *Wolbachia*. Note that in these simulations we assume the same mosquito populations for both genera. Typically the density of *Ae*. *aegypti* mosquitoes is much greater than the density for *Ae*. *albopictus* in urban areas, while the opposite is true in wooded rural areas. In this study, we only considered one mosquito genus at a time. When both mosquitoes are present, then the predictions will depend on the total mosquito population. As a first order approximation, the results are interpolated based on the fraction of each mosquito genus.

### The reproduction numbers

**Fig 3:**
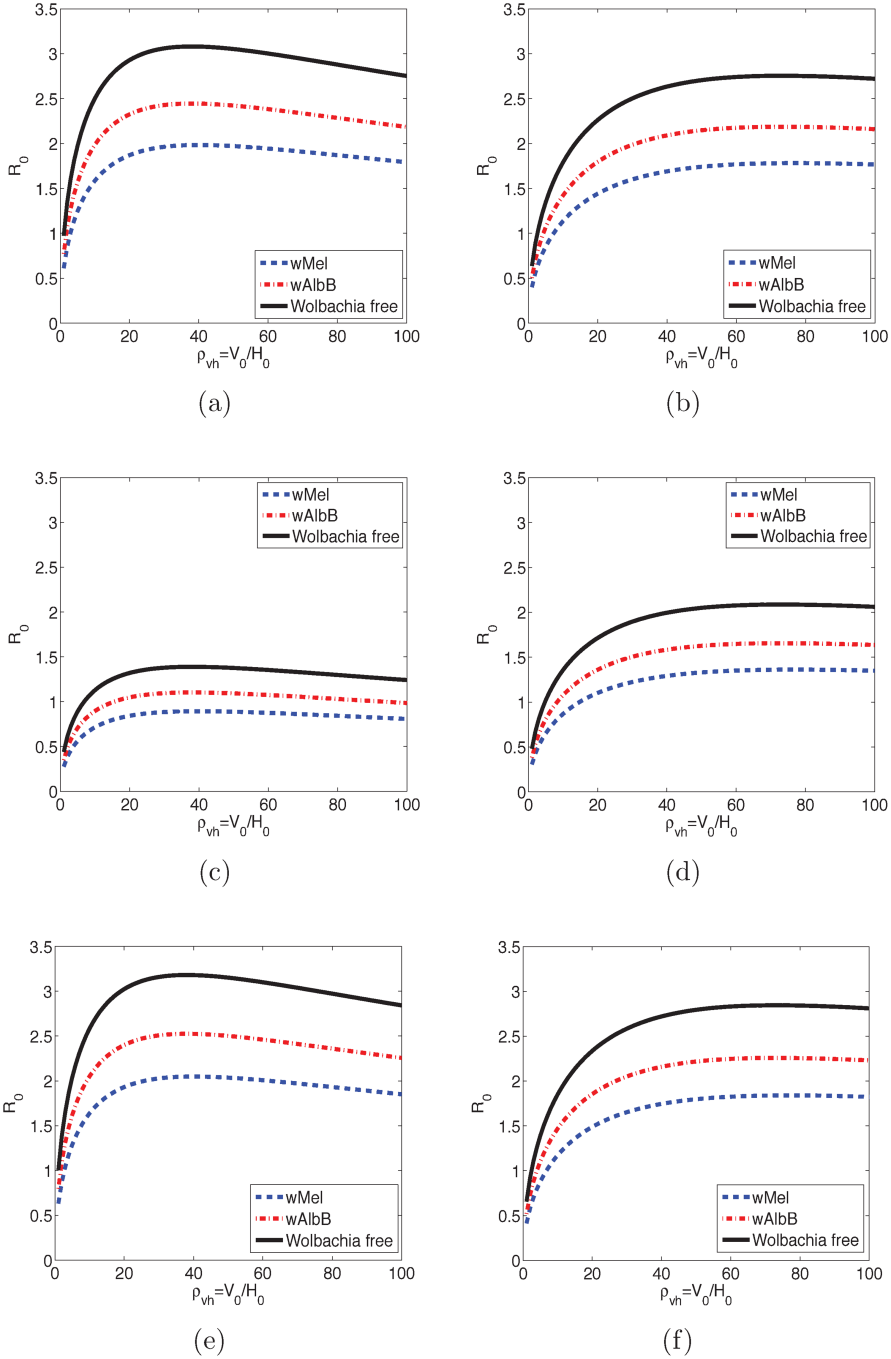
$R_{0}$ changes with the increase of initial number of mosquitoes per person. $R_{0}$ increases when few mosquitoes bite a person, and eventually decreases when there are many more mosquitoes than humans, *ρ*_*vh*_ > 40. In all cases, $R_{0}$ for infection transmitted by *Wolbachia*-free mosquitoes is the largest, followed by $R_{0}$ for infection transmitted by wAlbB-infected mosquitoes, and $R_{0}$ for infection transmitted by wMel-infected mosquitoes is the smallest. (a) $R_{0}$ for DENV transmitted by *Ae*. *aegypti* mosquitoes. (b) $R_{0}$ for DENV transmitted by *Ae*. *albopitus* mosquitoes. (c) $R_{0}$ for CHIKV transmitted by *Ae*. *aegypti* mosquitoes. (d) $R_{0}$ for CHIKV transmitted by *Ae*. *albopictus* mosquitoes. (e) $R_{0}$ for ZIKV transmitted by *Ae*. *aegypti* mosquitoes. (f) $R_{0}$ for ZIKV transmitted by *Ae*. *albopictus* mosquitoes.

The reproduction numbers depend on the ratio of mosquitoes to humans. The model predictions are scaled for all populations with the same *ρ*_*vh*_ = *V*_0_/*H*_0_, *V*_0_ is the initial total number of mosquitoes, and *V*_0_ = *K*_*v*_. In Fig 3, the reproduction numbers are plotted as *ρ*_*vh*_ varies from a ratio of *ρ*_*vh*_ = 1, with an equal number of mosquitoes as humans, to *ρ*_*vh*_ = 100, with 100 times more mosquitoes than humans. Infecting mosquitoes with *Wolbachia* can reduce $R_{0}$ over the full range of mosquito to human ratios.

When only few mosquitoes are present, then $R_{0}<1$ for all the diseases. As expected, $R_{0}$ increases as the number of mosquitoes increases, as more and more mosquitoes transmit the infection. When there are about 20 to 30 mosquitoes per human, then $R_{0}$ slowly decreases as the biting rate for the mosquitoes decreases. The rate of decrease depends upon the specific biting Eq (3) being used in the model.

Table 4 lists the basic reproduction number computed with the baseline parameters. For this case, the $R_{0}$ of DENV (ZIKV, or CHIKV) transmitted by *Wolbachia*-free mosquitoes is the highest, followed by $R_{0}$ of DENV (ZIKV, or CHIKV) transmitted by mosquitoes infected with wAlbB strain of *Wolbachia*, and $R_{0}$ for mosquitoes carrying wMel strain is the smallest. The basic reproduction number for ZIKV is the largest in all cases. The basic reproduction number of DENV is greater than the basic reproduction number of CHIKV for mosquitoes carrying the same strain of *Wolbachia* or *Wolbachia*-free mosquitoes.

**Table 4:** The basic reproduction number for the parameter values in Table 2. $R_{0}$ for ZIKV is the largest. For any strains of *Wolbachia*, $R_{0}$ for ZIKV is still greater than one. For the same virus, $R_{0}$ is the largest for *Wolbachia*-free mosquitoes, and second largest for wAlbB-infected mosquitoes.

*Wolbachia*	*Ae*. *aegypti*			*Ae*. *albopictus*		
	DENV	ZIKV	CHIKV	DENV	ZIKV	CHIKV
wMel	1.87	1.93	0.84	1.44	1.49	1.10
wAlbB	2.32	2.40	1.05	1.80	1.85	1.36
*Wolbachia*-free	2.93	3.02	1.32	2.26	2.34	1.71

**Fig 4:**
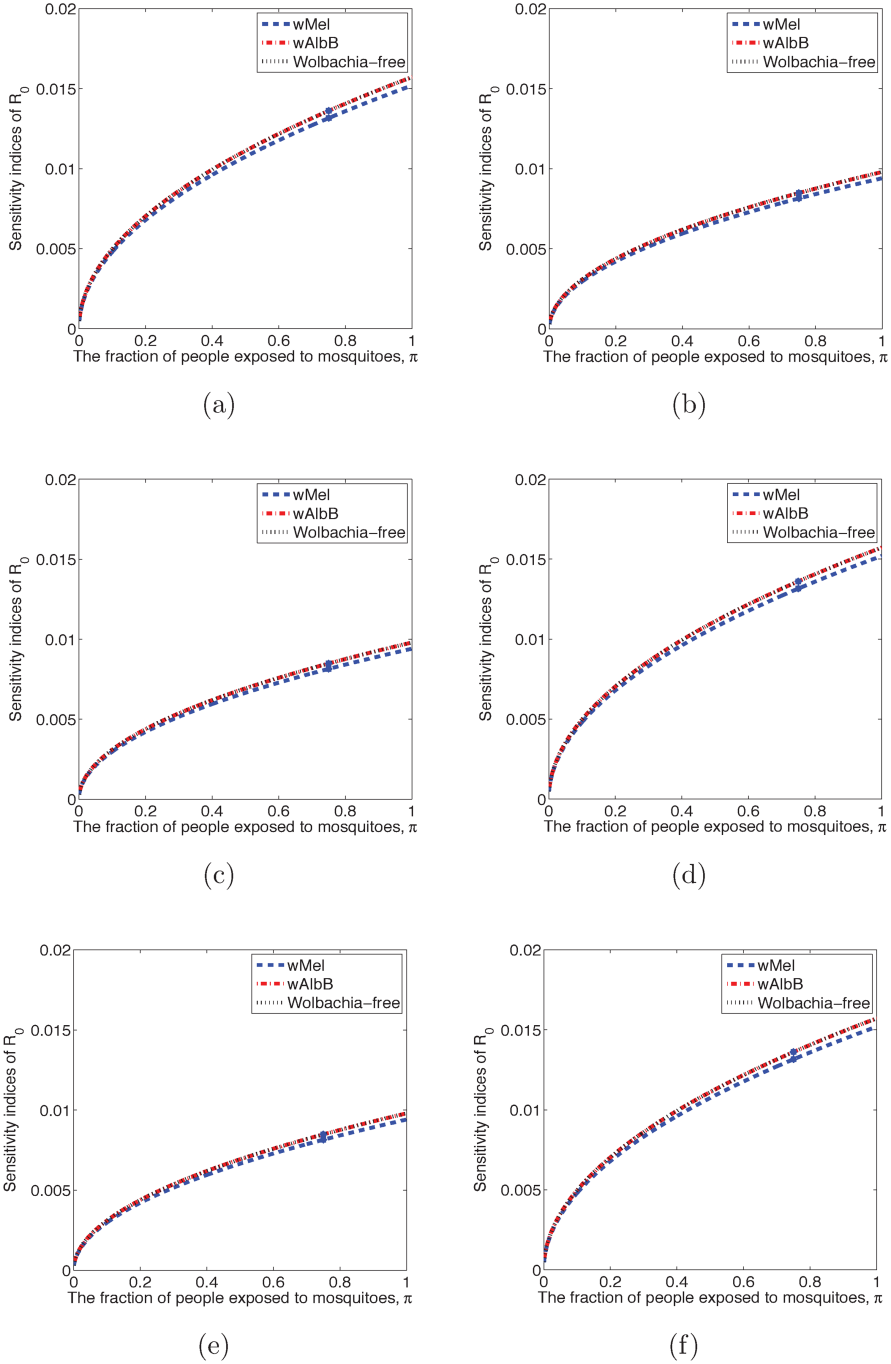
Sensitivity indices of $R_{0}$ changing with the fraction of people exposed to mosquitoes, *π*, ranging from 0 to 1. (a) Sensitivity indices of $R_{0}$ for DENV transmitted by *Ae*. *aegypti* mosquitoes. (b) Sensitivity indices of $R_{0}$ for DENV transmitted by *Ae*. *albopitus* mosquitoes. (c) Sensitivity indices of $R_{0}$ for CHIKV transmitted by *Ae*. *aegypti* mosquitoes. (d) Sensitivity indices of $R_{0}$ for CHIKV transmitted by *Ae*. *albopictus* mosquitoes. (e) Sensitivity indices of $R_{0}$ for ZIKV transmitted by *Ae*. *aegypti* mosquitoes. (f) Sensitivity indices of $R_{0}$ for ZIKV transmitted by *Ae*. *albopictus* mosquitoes.

The basic reproduction number is a function of the baseline parameters. Others may come up with different baseline values for different outbreaks. The readers can estimate the model response to different baseline values of a parameter using the sensitivity indices in Table 5. The relative sensitivity index of the quality of interest, *q*, with respect to the parameter of interest, *p* is

$$S_{p}^{q}\;≔\frac{p^{*}}{q^{*}}\times \frac{\partial q}{\partial p}{|}_{p=p^{*}}=\frac{\theta_{q}}{\theta_{p}},\;\;$$

as described in [23], where the notation p* indicates that a variable is evaluated at the model baseline values. For example, $S_{\pi }^{R_{0}}=0.0136$ for DENV transmitted by wAlbB-infected *Ae*. *aegypti* mosquitoes, if we reduce *π*, by 10%, then $R_{0}$ will be reduced by 0.00136, since −0.1 × 0.0136 = −0.00136. Sensitivity indices of $R_{0}$ varying with the fraction of people exposed to mosquitoes are shown in Fig 4.

**Table 5:** Sensitivity indices of $R_{0}$ for the Model (1) at the baseline parameter values in Tables 2 and 3. As can be seen from the table, for *Ae*. *aegypti* mosquitoes, the most sensitive parameter is the mosquito death rate, *μ*_*v*_. However, for *Ae*. *albopictus* mosquitoes, the most sensitive parameter is the mosquito biting rate, *σ*_*v*_, and the least sensitive parameter is the incubation rate for humans, *ν*_*h*_.

Parameter	DENV		DENV		ZIKV		ZIKV		CHIKV		CHIKV	
	*Ae*. *aegypti*		*Ae*. *albopictus*		*Ae*. *aegypti*		*Ae*. *albopictus*		*Ae*. *aegypti*		*Ae*. *albopictus*	
	wAlbB	wMel	wAlbB	wMel	wAlbB	wMel	wAlbB	wMel	wAlbB	wMel	wAlbB	wMel
*σ*_*v*_	+0.66	+0.67	+0.79	+0.79	+0.66	+0.67	+0.79	+0.79	+0.66	+0.67	+0.79	+0.79
*μ*_*v*_	-0.71	-0.72	-0.66	-0.67	-0.71	-0.72	-0.66	-0.67	-0.72	-0.73	-0.57	-0.58
*β*_*hv*_	+0.50	+0.50	+0.50	+0.50	+0.48	+0.48	+0.48	+0.48	+0.79	+0.79	+0.79	+0.79
*β*_*vh*_	+0.50	+0.50	+0.50	+0.50	+0.48	+0.48	+0.48	+0.48	+0.79	+0.79	+0.79	+0.79
*γ*_*h*_	-0.50	-0.50	-0.50	-0.50	-0.48	-0.48	-0.48	-0.48	-0.79	-0.79	-0.79	-0.79
*σ*_*h*_	+0.34	+0.33	+0.21	+0.21	+0.33	+0.32	+0.21	+0.20	+0.55	+0.53	+0.34	+0.33
*ν*_*v*_	+0.21	+0.22	+0.16	+0.17	+0.20	+0.21	+0.16	+0.17	+0.35	+0.37	+0.11	+0.12
*H*_0_	-0.16	-0.17	-0.29	-0.29	-0.15	-0.16	-0.28	-0.28	-0.25	-0.26	-0.45	-0.46
*V*_0_	+0.16	+0.17	+0.29	+0.29	+0.15	+0.16	+0.28	+0.28	+0.25	+0.26	+0.45	+0.46
*π*	+0.0136	+0.0132	+0.0085	+0.0081	+0.0141	+0.0136	+0.0088	+0.0084	+0.0086	+0.0083	+0.0054	+0.0052
*μ*_*h*_	-0.0007	-0.0007	-0.0007	-0.0007	-0.0007	-0.0007	-0.0007	-0.0007	-0.0010	-0.0010	-0.0010	-0.0010
*ν*_*h*_	+0.0003	+0.0003	+0.0003	+0.0003	+0.0003	+0.0003	+0.0003	+0.0003	+0.0003	+0.0003	+0.0003	+0.0003

**Fig 5:**
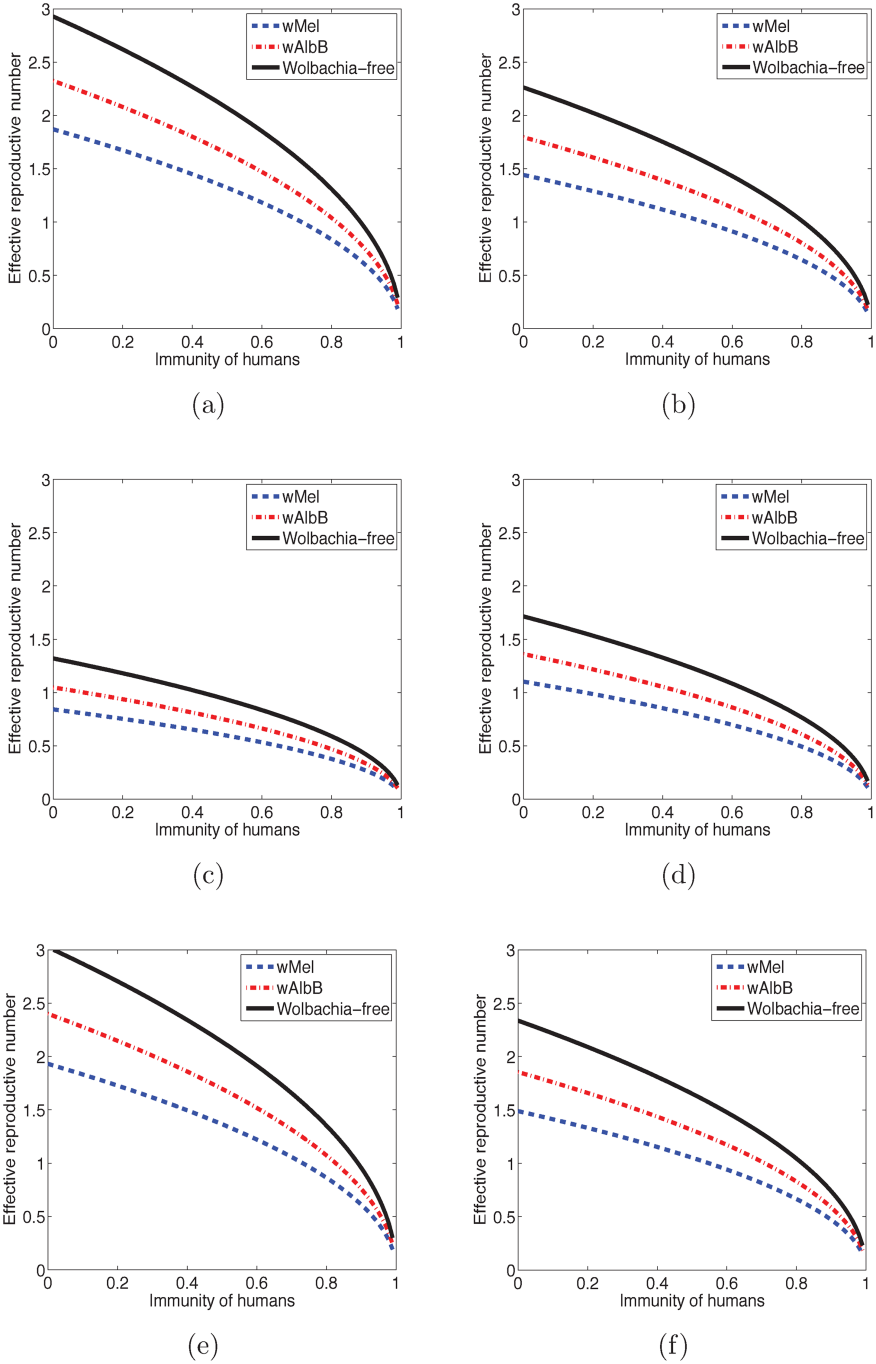
Initial effective reproduction number, $R_{eff}\left(0\right)$ changing with immunity in humans. The $R_{eff}\left(0\right)$ decreases as the immunity in humans increases and *Ae*. *aegypti* are infected with *Wolbachia*. This indicates that even though infecting the mosquitoes with *Wolbachia* might not reduce the initial $R_{0}$ to be less than one, it could be an effective strategy in future seasonal outbreaks to bring $R_{eff}$ to be less than one when part of the population is immune. (a) $R_{eff}\left(0\right)$ for DENV transmitted by *Ae*. *aegypti* mosquitoes. (b) $R_{eff}\left(0\right)$ for DENV transmitted by *Ae*. *albopictus* mosquitoes. (c) $R_{eff}\left(0\right)$ for CHIKV transmitted by *Ae*. *aegypti* mosquitoes. (d) $R_{eff}\left(0\right)$ for CHIKV transmitted by *Ae*. *albopictus* mosquitoes. (e) $R_{eff}\left(0\right)$ for ZIKV transmitted by *Ae*. *aegypti* mosquitoes. (f) $R_{eff}\left(0\right)$ for ZIKV transmitted by *Ae*. *albopictus* mosquitoes.

The effective reproduction number depends on the fraction of humans who are immune to the infection. Fig 5 shows the effective reproduction number varying with the immunity of humans, assuming that all mosquitoes are susceptible and the initial total mosquito population is *V*_0_. The effective reproduction number decreases with the increase of the immunity of humans. When all humans are immune to the disease, then the effective reproduction number is the same as the basic reproduction number.

**Fig 6:**
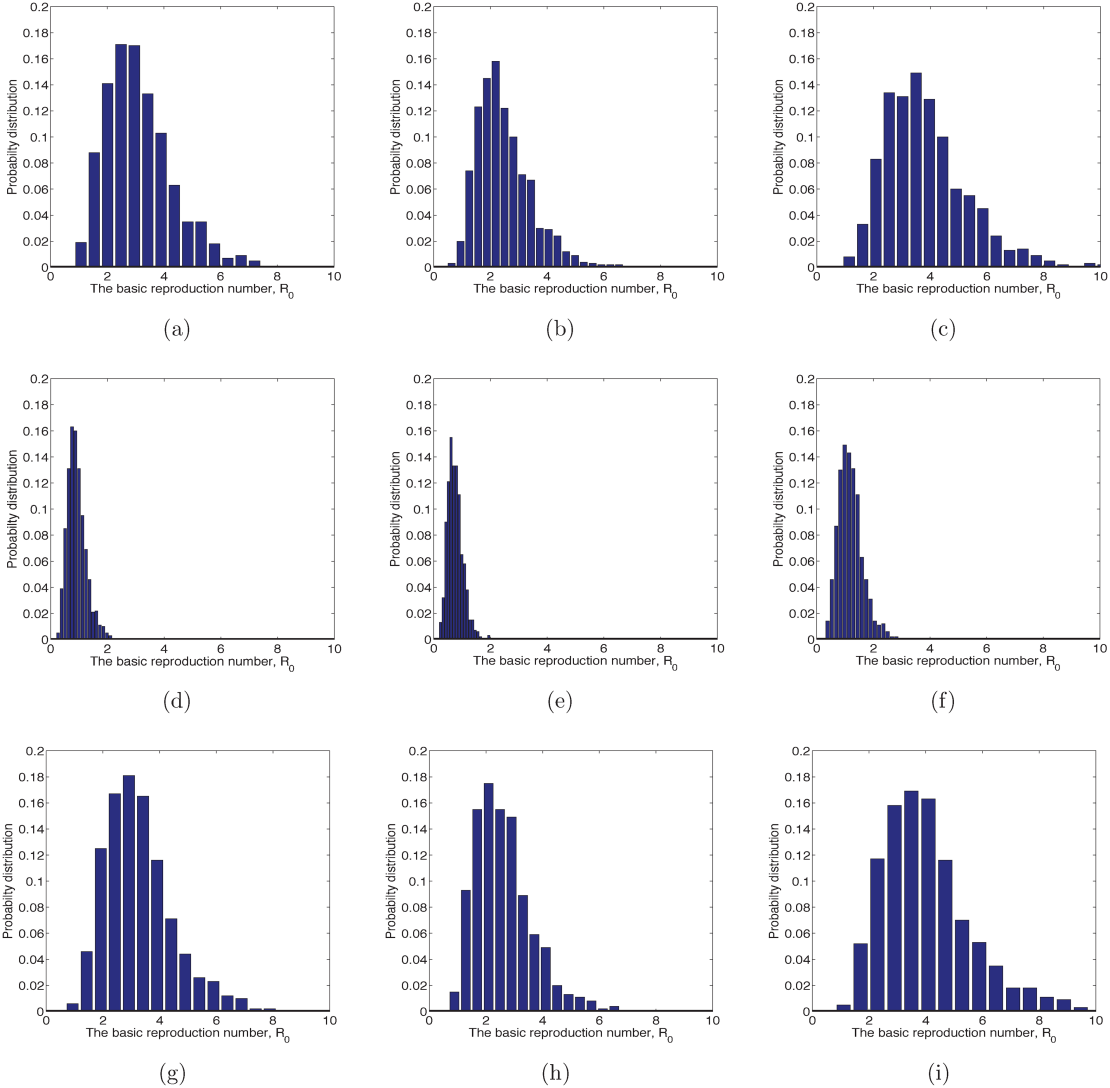
Histogram for $R_{0}$. Histograms from 1000 realizations of $R_{0}$ for DENV, ZIKV and CHIKV when *Wolbachia*-infected and *Wolbachia*-free *Ae*. *aegypti* mosquitoes. The baseline parameters in Table 2 were sampled from a triangular distribution having the mode at the baseline values and vanishing at the edges of the feasibility region. The mean and median of Zika are the largest. (a) When DENV is transmitted by wAlbB-infected mosquitoes, median and mean of $R_{0}$ are 2.94 and 3.13, respectively. (b) When DENV is transmitted by wMel-infected mosquitoes, median and mean of $R_{0}$ are 2.30 and 2.48, respectively. (c) When DENV is transmitted by *Wolbachia*-free mosquitoes, median and mean of $R_{0}$ are 3.65 and 3.84, respectively. (d) When CHIKV is transmitted by wAlbB-infected mosquitoes, median and mean of $R_{0}$ are 0.88 and 0.92, respectively. (e) When CHIKV is transmitted by wMel-infected mosquitoes, median and mean of $R_{0}$ are 0.73 and 0.76, respectively. (f) CHIKV transmitted by *Wolbachia*-free mosquitoes, median and mean of $R_{0}$ are 1.14 and 1.19, respectively. (g) When ZIKV is transmitted by wAlbB-infected mosquitoes, median and mean of $R_{0}$ are 3.10 and 3.28, respectively. (h) When ZIKV is transmitted by wMel-infected mosquitoes, median and mean of $R_{0}$ are 2.45 and 2.62, respectively. (i) When ZIKV is transmitted by *Wolbachia*-free mosquitoes, median and mean of $R_{0}$ are 3.79 and 4.03, respectively.

Previous examples kept most of the parameters at their baseline values. If we allow all the parameters to vary over the entire feasible sampling space, we will obtain a distribution for $R_{0}$. The distribution for $R_{0}$ is a function of the distributions for the model parameters as they vary within their allowed ranges. We assumed a triangular distribution that vanishes at the endpoints of the feasible region and has the mode at the baseline values in Table 2. If we had assumed that the distribution was uniform over the range, where the parameter was just as likely to be at the upper or lower bound as our best guess (the baseline case), then the ranges for the reproduction numbers in Fig 6 will not change, but the distributions will have fatter tails.

The histograms of the distribution for $R_{0}$ for DENV, ZIKV, and CHIKV transmitted by *Ae*. *aegypti* are shown in Fig 6. The means and medians of $R_{0}$ for wMel-infected mosquitoes are the smallest. $R_{0}$ for ZIKV is the largest, followed by DENV, and then CHIKV. In a similar analysis for *Ae*. *albopictus*, $R_{0}$ is the largest for ZIKV, followed by CHIKV, and the least is DENV. This is in agreement with the analysis where we varied the parameters one at a time over their feasible ranges.

### Endemic equilibrium analysis

If *Wolbachia* is successful in reducing the spread of the viruses, then there will be more people uninfected at the EE. In Table 6, the fraction of humans still susceptible at the EE for wMel-infected mosquitoes is the largest, while the susceptibility of *Wolbachia*-free mosquitoes is the smallest when a certain disease is transmitted by a certain genus of mosquitoes. For *Ae*. *aegypti* infected with the same strain of *Wolbachia*, the percentage of humans susceptible to CHIKV is higher than the percentage of humans susceptible to DENV or Zika. For *Ae*. *albopictus* infected with the same strain of *Wolbachia*, the percentages of humans susceptible to DENV and ZIKV are higher than the percentage of humans susceptible to CHIKV. Although the wMelPop-infected mosquitoes are the most effective in stopping an epidemic, it is unrealistic to consider a fully infected wild population of wMelPop-infected mosquitoes. Hence, we did not include the analysis for wMelPop strain of *Wolbachia*. This coefficient for effectiveness computed using Eq (7) listed in Table 7 shows that wMel is significantly more effective than wAlbB in reducing the number of infections in simulations with the baseline parameters in Table 2.

**Table 6:** The fraction of susceptible humans at the endemic equilibrium. A larger susceptibility means fewer people are infected at the endemic equilibrium.

*Wolbachia*	*Ae*. *aegypti*			*Ae*. *albopictus*		
	DENV	ZIKV	CHIKV	DENV	ZIKV	CHIKV
wMel	0.21	0.21	0.42	0.36	0.36	0.25
wAlbB	0.14	0.14	0.27	0.23	0.23	0.16
*Wolbachia*-free	0.09	0.09	0.17	0.15	0.15	0.10

**Table 7:** The coefficient for effectiveness, *κ*. This coefficient measures the relative reduction in the total number of humans infected with DENV, ZIKV, or CHIKV transmitted by wMel- and wAlbB-infected mosquitoes, with respect to the number of humans infected with *Wolbachia*-free mosquitoes, by *t* = 700. Note that wMel is more effective than wAlbB in each case.

*Wolbachia*	*Ae*. *aegypti*			*Ae*. *albopictus*		
	DENV	ZIKV	CHIKV	DENV	ZIKV	CHIKV
wMel	0.04	0.03	0.95	0.17	0.15	0.61
wAlbB	0.01	0.01	0.65	0.04	0.03	0.18

## Discussion

A mosquito infected with the *Wolbachia* bacteria is less capable of transmitting DENV, ZIKV, and CHIKV, and one of the leading new mitigation strategies is to fight the spread of these viral infections by releasing *Wolbachia*-infected mosquitoes. We quantified the impact of wAlbB, wMel, and wMelPop strains of *Wolbachia* in reducing the transmission of CHIKV, DENV, and ZIKV. The model accounts for reduced fitness of the *Wolbachia*-infected mosquitoes, reduced ability of transmitting viruses, and the behavior changes of infected individuals caused by the infection. Because people infectious with DENV and CHIKV are more likely to have serious symptoms, we assumed that the people infectious with these viruses were less likely to be bitten by mosquitoes than the people infectious with ZIKV. The behavior changes of humans have significant impacts on the model predictions and, unfortunately, is often let out of most vector-borne disease models.

The baseline model parameters are estimates for the general population and our best guess from the literature. The relative sensitivity indices for these parameters (Table 5) can be used to predict how slightly different assumptions about these input parameters will change the basic reproduction number. The relative sensitivity analysis quantified the relative importance of the model parameters on the model predictions and can be used to quantify the importance of obtaining more accurate data to reduce the parameter uncertainty and improve the model’s predictability.

Over the entire range of parameter values, our simulation results show that the $R_{0}$ for wMel-infected mosquitoes is the smallest. For *Ae*. *aegypti*, $R_{0}$ for ZIKV is the largest, followed by DENV, then CHIKV. In a similar analysis for *Ae*. *albopictus*, $R_{0}$ is the largest for ZIKV, followed by DENV, then CHIKV. This is in agreement with the analysis where we varied the parameters one at a time over their feasible range. Our simulation results show that the wMel strain is more effective in controlling these viruses than wAlbB strain in all of the situations we tested. We find that for the same mosquito densities, *Ae*. *aegypti* is more effective than *Ae*. *albopictus* in transmitting DENV and ZIKV, while the opposite is true for CHIKV.

The results are based on the simulations with the parameter values available in current literature, which may vary for different locations at different times. Our model is a general model that can produce outputs for a specific location, once the data for the location are available to parameterize the model.

Comparisons of the model predictions for *Ae*. *aegypti* versus *Ae*. *albopictus* must take into account the ratio of mosquitoes to humans. $R_{0}$ is sensitive to this ratio and the density of *Ae*. *aegypti* mosquitoes is typically higher in urban areas than in rural areas, while the opposite is true for *Ae*. *albopictus* mosquitoes [37]. When there are few mosquitoes per human, then $R_{0}<1$. As the number of mosquitoes increases, then $R_{0}$ quickly increases to be greater than one. As the number of mosquitoes per human becomes very large, $R_{0}$ eventually decreases in our model where the number of times that humans allow themselves to be bitten is limited to a maximum number of times per day. The rate of decrease depends upon the specific biting rate in Eq (3) being used in the model.

Although wMelPop-infected mosquitoes do not transmit these viruses, the increased death rate of wMelPop-infection has a high fitness cost. It is difficult for wMelPop-infected mosquitoes to survive in the wild mosquito populations because a much larger number of wMelPop-infected mosquitoes needs to be released in order to sustain in the wild mosquito population.

The analysis of the basic reproduction number assumes that when the infections first enter a population, then everyone is fully susceptible to the infection. We derived the effective reproduction number for when the host population is partially immune to new infections, perhaps due to a previous epidemic. The effective reproduction number increases with the susceptibility of humans. When more people are immune to DENV and CHIKV than ZIKV, as happened in the 2015 Zika epidemic, then the numbers of dengue and Chikungunya cases tend to be stable, while the number of Zika cases exploded. Hence, the susceptibility of the human population must be taken into account in future seasonal outbreaks. Our analysis quantified how $R_{eff}\left(t\right)$ depends upon a fraction of the population being immune to the infection in a vector-host transmission model.

There are ongoing efforts for releasing *Wolbachia*-infected mosquitoes in the wild to fight against the spread of these viral infections. *Wolbachia*-infected mosquitoes could contribute to the reduction of transmission instead of elimination. Besides, the number of *Wolbachia*-infected mosquitoes released has to exceed the threshold and continual introductions are required. The real-world thresholds for sustaining an epidemic will be greater than the threshold estimates derived for ideal conditions where there is a homogenous population of infected and uninfected mosquitoes.

These field tests suggest that the spatial heterogeneity of the populations must be considered before this model will be appropriate to help guide policy decisions. Also, our simulations are based on an environment of *Wolbachia*-infected mosquitoes where most of the wild mosquitoes are infected with *Wolbachia*. When introducing the wMel or wAlbB strains of *Wolbachia* into a wild mosquito population, it may take several weeks or months for it to reach the equilibrium level and may require several introductions [22]. Furthermore, the model parameter values are based on average estimates from the literature, and not the parameters for a specific location. Before this model can be applied to a specific location, then model parameters, such as the average number of mosquitoes per person, must be estimated for this location.

In future studies, we will couple the model for the spread of *Wolbachia* [22] with the disease transmission model [23] to evaluate effectiveness of this approach for the situations where the mosquito population is only partially infected with *Wolbachia* and consider new human arrivals including people who are immune and infectious.
